# Quality of life in HIV/AIDS

**DOI:** 10.4103/2589-0557.74971

**Published:** 2010

**Authors:** K. H. Basavaraj, M. A. Navya, R. Rashmi

**Affiliations:** Department of Dermatology, Venereology and Leprosy, JSS Medical College, JSS University, Mysore, Karnataka, India

**Keywords:** AIDS, antiretroviral therapy, anxiety, coping, depression, HIV, psychosocial, quality of life, social support

## Abstract

Given the longevity achievable with current prophylactic and therapeutic strategies for persons with HIV infection, quality of life (QOL) has emerged as a significant medical outcome measure, and its enhancement has an important goal. This review highlights the relevance and complexity of physical, psychological, and social factors as determinants of health-related quality of life in HIV-infected persons. Existing data suggest that physical manifestations, antiretroviral therapy, psychological well-being, social support systems, coping strategies, spiritual well-being, and psychiatric comorbidities are important predictors of QOL in this population. Consequently, the impact of HIV infection on the dimensions of QOL, including physical and emotional well-being, social support systems, and life roles, has emerged as a key issue for persons infected with HIV.

## INTRODUCTION

Quality of life (QOL) is a term that is popularly used to convey an overall sense of well-being and includes aspects such as happiness and satisfaction with life as a whole. World Health Organization has defined QOL as “individuals’ perceptions of their position in life in the context of the culture and value systems in which they live and in relation to their goals, standards, expectations and concerns.”[1] With the recent advances in clinical tests and treatments for those suffering from human immunodeficiency virus (HIV)/acquired immunodeficiency syndrome (AIDS), the survival of these patients has been increased and their QOL has become an important focus for researchers and healthcare providers.[2] Since the discovery of HIV at in beginning of the 1980s, HIV/AIDS has been one of the greatest health problems in the world.[3] HIV/AIDS places an increasing burden on the health of the population, and causes further socioeconomic problems for individuals, families, communities, and governments in many countries.[4,5] HIV is increasingly considered a chronic disease. For a person living with HIV, this means having to cope with a range of HIV-related symptoms for extended periods. Symptoms may be related to the infection itself, comorbid illnesses, or iatrogenic effects from HIV-related medications.[6,7] Many of the HIV patients struggle with numerous social problems such as stigma, poverty, depression, substance abuse, and cultural beliefs which can affect their QOL not only from the physical health aspect, but also from mental and social health point of view and cause numerous problems in useful activities and interests of the patients.[8] Assessing health-related quality of life (HRQOL) is useful for documenting the patients’ perceived burden of chronic disease, tracking changes in health over time, assessing the effects of treatment and quantifying the return on health care investment.[9] This article reviews recent findings concerning the different aspects of QOL in HIV patients.

**Figure 1:**
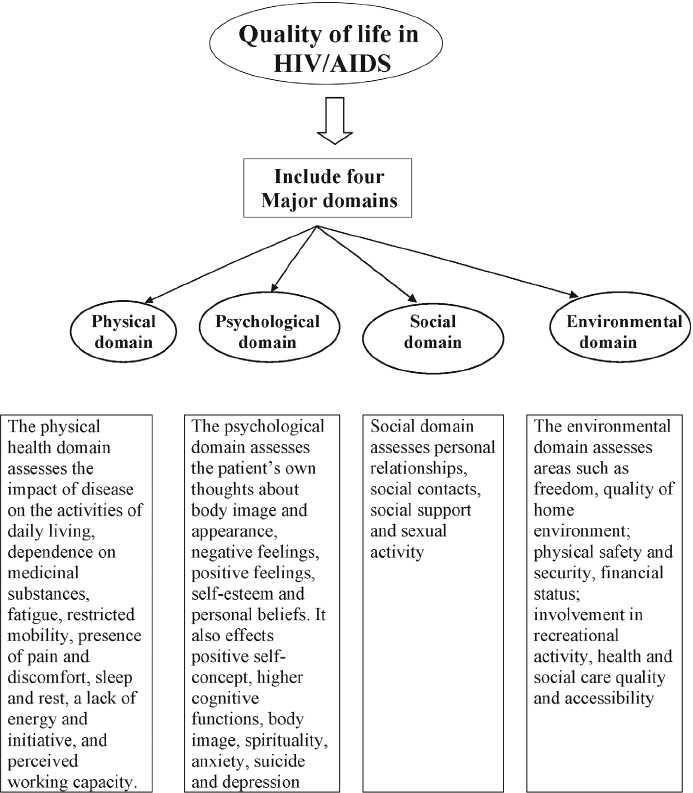
Quality of life – major domains

Several factors associated with better QOL among HIV-infected patients have been reported in the international literature, and mainly, the impact of HIV on QOL falls under four major domains [Figure 1]. Sociodemographic characteristics such as male gender[10] younger age,[11] higher socioeconomic status,[12] and employment[12] have been associated with improvement in QOL. Other variables such as lower HIV viral load,[13] greater CD4+ cell count,[11,13,14] fewer or less bothersome HIV symptoms,[15] and higher levels of hemoglobin[16] have been shown to be important clinical/immunological indicators of better QOL. In addition, patients with no difficulty in taking medications,[11] those using regimens with a lower number of pills,[11] and those more adherent to antiretroviral therapy (ART)[10,12,13] tend to have improved QOL following the start of treatment.

Many people living with HIV/AIDS find it challenging to attend to daily tasks of living, participate in moderate to vigorous physical activities, or have sufficient energy or vitality to engage in an active social life while managing HIV/AIDS. Fatigue or low energy has been associated with both physical and psychological morbidity[17] and poor QOL[18] in persons with HIV/AIDS. In addition, fatigue and a CD4 T-cell count less than 500 are associated with physical limitations and disability.[19] Among HIV - positive patients, disease progression is related to decreasing energy and increasing difficulties with daily activities and pain.[20]

HIV/AIDS coping by disengagement or avoidance was associated with greater health-related stress.[21] In addition, poor social functioning may be associated with greater use of avoidance coping strategies such as withdrawal and conflictual social interactions. Social isolation and conflictual social interactions have been shown to increase stress, resulting in poorer overall social functioning.[22] People living with HIV/AIDS who increase their use of avoidance coping strategies such as behavioral disengagement and self-distraction as well as their use of alcohol and drugs may have poorer physical and social functioning.[23] HIV-infected individuals with a history of injected drug use, especially those with more severe drug problems, report health-related QOL.[24] Complex role functioning (i.e., career, housework, and educational pursuits) has been shown to be even more limited than physical functioning in people with HIV/AIDS,[25] suggesting that coping by avoidance may directly reduce an individual’s productivity.

### Impact of antiretroviral therapy

The development of combined ART has shifted the perception of HIV/AIDS from a fatal to a chronic and potentially manageable disease. ART is capable of improving survival, reducing the occurrence of HIV-related opportunistic infections, and improving the patients’ QOL.[26]

### Impact of psychosocial factors

The impact of social, psychological, and spiritual factors on QOL in HIV infection has been well recognized.[27–28] Stressful events and social support were related to HIV-1 disease progression to AIDS.[29] Research on the psychosocial aspects of HIV-positive status shows that living with HIV is associated with a large measure of stress and depression.[30]

### Social support

Social support for patients with HIV/AIDS has shown a strong potential to influence HRQOL. The three major components of social support are emotional, tangible, and informational support.[31] Distinction among the different types of social support is relevant, since their functions may not be necessarily interchangeable. Emotionally sustaining function of social support, which serves to fulfill and gratify one’s need for nurturance, belonging, and alliance, is well recognized to buffer stress in non-HIV settings. At least two studies have reported that emotionally sustaining support was considered more desirable and was more often used than other forms of support.[32]

### Coping

Coping is another variable influencing QOL. Pearlin and School have defined coping as the cognitive and behavioral effort made to tolerate, reduce, or master demands that challenge or exceed a person’s resources. Individuals who confronted stress with problem-solving and behavior-modifying approaches had a significantly better QOL than those not using such coping skills.[22] It has been proposed that education and behaviorally oriented interventions that enhance problem solving and active decision making are likely to be more beneficial than emotionally supportive interventions that encourage passive acceptance of the illness. Coping by denial (avoidance) was associated with a significantly lower QOL in a previous study. Although denial has been shown to be an effective coping method in non-HIV settings, the preponderance of studies in HIV settings has suggested otherwise.[22] Denial has been shown to correlate with low self-esteem and depression in HIV patients. Indeed, coping by denial may be an expression of helplessness, anger, or depression, and these patients may, in fact, be in need of psychological intervention.[22]

### Spirituality

Spirituality is an important contributor to feelings of well-being. Spirituality among HIV-infected individuals was perceived as a bridge between hopelessness and meaningfulness in life.[33] Creating meaning and purpose in life more than religious experiences was found to correlate with psychological well-being in a large sample of African American men and women with HIV/AIDS.[29]

### Depression

Comorbid psychiatric illnesses, including depression, are common in HIV-infected patients.[34] The prevalence of depression in HIV-infected clinic populations has ranged from 22% to 38%.[34,35] Younger age, unemployment, lack of health insurance, low CD4+ cell counts, HIV-related symptoms, not having a partner, poor quality of social support, and use of noninjection drugs were significant predictors of depression in one study.[36] Patients with HIV infection who are older than 35 years are more likely to suffer from depression, anxiety, confusion, and fatigue. Insomnia, pain, and emotional control correlated with depression.[37] Physical limitations may also contribute to depression; after controlling for disease stage, physical symptoms, and CD4 cell counts, the degree of physical limitation in one study predicted depression.[38]

The impact of psychiatric comorbidities, specifically depression, on the HRQOL of patients with HIV disease has been well documented.[39–42] The presence of a major psychiatric disorder (independent of HIV-related disease progression) was associated with a negative impact on HRQOL dimensions of mental health, social functioning, and general health perceptions but not on physical health, role functioning, or pain.[41] A larger study showed that patients with comorbid mood disorders had significantly worse functioning and well-being than those without mood disorders.[41]

Treatment of depression in patients with HIV disease may not prolong life but can lower the risk of suicide and improve QOL, both directly and through increased adherence to complex medical regimens.[43]

### HIV and unemployment

As people with HIV/AIDS adjust to living with a chronic illness, many new challenges emerge; among them are issues of occupational functioning and employment.[44] For working individuals, employment provides not only financial benefits but may also be a source of structure, social support, role identity, and meaning.[12]

Adults with HIV infection and AIDS often struggle with vocational dilemmas. Unlike acute medical conditions in which patients may return to predisease levels of functioning after treatment, patients with HIV infection must frequently adapt to an unpredictable illness course.[45] Even when physical health is stable, fear and uncertainty about how HIV disease will affect economic, occupational, and healthcare security complicate vocational decision making.[45] While some leave the workforce and receive disability benefits, others remain employed to varying degrees. Those who do work often find their occupational functioning limited by HIV-specific factors such as episodic illness, fatigue, physical and cognitive limitations, medication schedules and side effects, and frequent medical appointments.[12]

Previous research has demonstrated that unemployed individuals generally report more depression, anxiety, social isolation, and low self-esteem than employed individuals.[46] In the HIV/AIDS literature, studies that have incorporated employment as a variable of interest have yielded similar findings. Kelly *et al*.[34] found that unemployment was one of several factors associated with suicidal ideation in HIV-seropositive patients. In a study of psychosocial vulnerability in HIV-seropositive gay men, Dickey *et al*.[45] reported that younger men who lacked full-time employment were at greater risk for psychiatric symptoms and syndromal depression. Finally, Swindells *et al*.[12] found that employment was one of several factors associated with improved QOL.

### Suicidal ideation, suicide attempts, and HIV infection

Suicide, attempted suicide, and suicidal ideation are complex clinical issues associated with life-threatening conditions such as HIV infection. Suicide in persons with HIV infection/AIDS has been reported in most cases to be associated with a concomitant psychiatric disorder.[46] The risk of suicide may extend to those fearful of contracting HIV infection[40] as well as the family and partners of those infected.[41] Elevated lifetime rates of affective disorders (particularly major depression) and substance use disorders have been reported in studies of HIV-positive men.[32] Furthermore, certain patterns of behavior associated with the risk of acquiring HIV infection (e.g., injection drug use) may be associated with higher levels of suicidal ideation and psychiatric disorders.

Patterns of attempted suicide and suicidal thoughts may differ throughout the progression of HIV infection. There are at least two high-risk periods: (1) the initial 6 months after diagnosis of infection with HIV and (2) the onset of physical complications of AIDS. The period of greater risk may be the first 3 months.

## CONCLUSION

Quality of life is a multidimensional concept whose definition and assessment remains controversial. HIV/AIDS represents a high economic impact from society point of view.

Overall self-perception of QOL has been shown to be a useful screening item for assessing global QOL. QOL relates both to adequacy of the material circumstances and to personal feelings about these circumstances. As health is generally cited as one of the most important determinants of overall QOL, it has been suggested that QOL may be uniquely affected by specific disease process such as AIDS. There is lack of clarity in defining QOL and concomitant operational difficulties in it but still there is urgency in evaluating the QOL in HIV-infected individuals. Future studies should encompass the evaluation of more determinants of QOL in HIV/AIDS. The constellations of HIV-related symptoms negatively affect the QOL for people living with HIV infection. Effective management of symptoms is important for improving QOL and potentially for maintaining a complicated daily regimen of ART. As HIV disease is among the most devastating of illnesses, having multiple and profound effects upon all aspects of life, hence the evaluation of QOL is very important. Although research has suggested relationships among various psychosocial and spiritual factors, symptomatology, and physical health, much more research is still needed to document their potential influences on immune function, as well as health status, disease progression, and QOL among persons with HIV disease. It is also important to underline the role of consultation-liaison psychiatry in the diagnosis and treatment of HIV and AIDS. Stress management interventions for HIV-infected persons are a promising approach to facilitate positive adjustment. Additional research is needed to further evaluate the role of routine QOL assessment in patients who have HIV/AIDS.

## Multiple Choice Questions

Q.1. Health-related quality of life (HRQOL) is useful for documenting the following:

- Patient’s perceived burden of chronic disease,
- Tracking changes in health over time,
- Assessing the effects of treatment and quantifying the return on healthcare investment
- Educational background

Q.2. Which one is not a sociodemographic characteristic:

- Gender and younger age
- Higher socioeconomic status
- Employment
- Religion

Q.3. Coping by denial may be an expression of:

- Helplessness
- Anger
- Depression
- All of the above

Q.4. Treatment of depression in patients with HIV disease:

- May not prolong life
- Lower the risk of suicide and improve QOL
- Increase adherence to complex medical regimens
- All of the above

Q. 5. High-risk periods of HIV infection:

- The initial 6 months after diagnosis of infection with HIV
- Onset of physical complications of AIDS
- a and b
- None of the above

### Answers

Q.1. d. Educational background
Q.2. d. Religion
Q.3. d. All of the above
Q.4. d. All of the above
Q.5. c. a and b
